# Enhancing Titanium Surface Properties with Castor Oil-Based Polyurethane: A Sustainable and Low-Cytotoxicity Approach

**DOI:** 10.1021/acsomega.5c05701

**Published:** 2025-12-01

**Authors:** Jonathan Ferreira Costa, Maelson Sousa Nunes, José Ribeiro dos Santos Júnior, Thátila Wanessa Vieira de Sousa, Wanderson Gabriel Gomes de Melo, Napoleão Martins Argolo Neto, José Milton Elias De Matos, Jorge Felipe Lima Teixeira, e Ana Cristina Vasconcelos Fialho

**Affiliations:** † Postgraduate Program in Dentistry, Federal University of Piauí—UFPI, Teresina 64049-550, Brazil; ‡ Postgraduate Program in Chemistry, Federal University of Piauí—UFPI, Teresina 64049-550, Brazil; § Postgraduate Program in Materials Science and Engineering, Federal University of Piauí—UFPI, Teresina 64049-550, Brazil; ∥ Postgraduate Program in Technology Applied to Animals of Regional Interest, Federal University of Piauí—UFPI, Teresin 64049-550, Brazil; ⊥ Center for Evolutionary Hologenomics—Globe Institute, 4321 University of Copenhagen, Copenhagen DK 1165, Denmark

## Abstract

Introduction: Titanium implants are widely used in medical and dental applications due to their excellent mechanical properties, biocompatibility, and corrosion resistance. However, surface modifications are often necessary to enhance osseointegration and improve long-term clinical outcomes. In this context, polymeric coatings have gained prominence as a means of modifying the surface properties of titanium implants. Castor oil-based polyurethane (PU) represents a promising alternative due to its low cytotoxicity, biodegradability, and environmentally friendly synthesis process. This study aims to investigate the physicochemical and biological properties of castor oil-based PU coatings on titanium substrates, assessing their potential as a biomaterial for surface modification of titanium implants. Objectives: To produce and evaluate the physicochemical and biological properties of castor oil-based polyurethane (PU) coatings on titanium substrates, we focused on their potential as a biomaterial for surface modification of titanium implants. Materials and Methods: PU was synthesized from castor oil and characterized by Fourier transform infrared spectroscopy (FTIR) to confirm the synthesis. Thermal stability was analyzed by thermogravimetric analysis (TGA). Surface morphology was investigated by using scanning electron microscopy (SEM) and atomic force microscopy (AFM), while adhesion of PU to titanium was confirmed by X-ray photoelectron spectroscopy (XPS). Increased surface hydrophilicity, confirmed by contact angle tests, and the successful adhesion of polyurethane functional groups identified by XPS highlight the low cytotoxicity of the material, which was analyzed by in vitro cytotoxicity assays. Results: FTIR analysis confirmed the successful synthesis of PU. Thermal analysis demonstrated that the material remained stable up to 200 °C, with distinct degradation events beyond this temperature range, while SEM and AFM revealed increased surface roughness and porosity, enhancing cell adhesion and osseointegration potential. Contact angle measurements indicated improved hydrophilicity, and XPS confirmed the strong adhesion of PU to titanium. Cytotoxicity assays showed cell viability above 70%, suggesting no significant cytotoxic effect. Conclusion: Castor oil-based PU proved to be a viable and environmentally friendly alternative for titanium implant coatings, with promising physicochemical and biological properties. MTT assay results demonstrated no significant cytotoxic effect, suggesting the potential for future in vivo biocompatibility studies.

## Graphical abstract

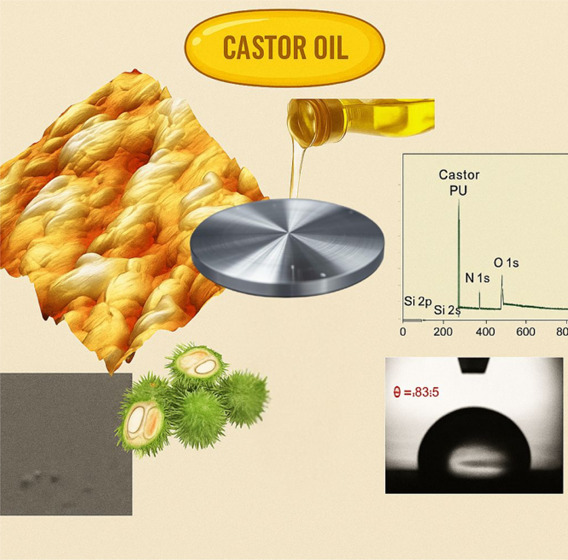

## 1 Introduction

Osseointegration refers to the direct and stable connection between bone tissue and the titanium surface (TS), crucial for the long-term success of dental implants.^1–3^ Histologically, it is defined by intimate contact between the bone and the implant, while clinically, it reflects the implant’s ability to remain functional and anchored in the bone over time. Titanium remains the most widely used metal for dental implants due to its excellent biocompatibility, mechanical strength, corrosion resistance, and proven osseointegration capabilities.^4^ However, the surface characteristics of titanium implants play a key role in modulating the biological response and influencing clinical outcomes.^5^

To enhance tissue integration, several physicochemical surface modifications have been proposed, aiming to improve protein adsorption, cell adhesion, proliferation, and osteogenic differentiation.^5–7^ Among these, topographical changes have been extensively studied, as surface roughness and microstructure significantly affect cellular behavior and bone apposition.^2,8,9^ Techniques such as sandblasting, acid etching, anodizing, and plasma spraying are commonly employed to achieve favorable surface textures.^5,8,9^ Furthermore, surface coatings with bioactive materials such as hydroxyapatite, bioactive glasses,^10^ or polymers have been explored to further optimize implant integration.^5,8^

Polyurethanes (PUs) have been widely explored for biomedical coatings due to their versatility, mechanical properties, and good interaction with biological environments. When synthesized from renewable sources, such as castor oil (*Ricinus communis*), PUs also represent an environmentally sustainable alternative to petroleum-based polymers.^11^ Castor oil-derived PU, in particular, combines desirable mechanical strength and wear resistance with low cytotoxicity and favorable interactions with biological tissues, making it especially suitable for biomedical applications.^12–15^

Unlike previous studies on conventional synthetic PUs, this work explores a castor oil-derived polyurethane, a fully renewable and environmentally friendly polymer, as a titanium coating. The study provides a comprehensive physicochemical and biological characterization of this sustainable alternative. In this context, the development and characterization of a castor oil-derived PU coating for titanium surfaces may offer a sustainable strategy for improving implant–tissue interactions while maintaining or improving the desirable physicochemical and biological performance of the implant system.

## 2 Materials and Methods

### 2.1 Production of Castor Oil Polyurethane

The synthesis of the polyurethane was carried out in a system with a controlled heating bath under constant stirring.^12,15–17^ With an initial temperature of 100 °C, castor oil (Mundo dos Óleos, Brasília, Brazil) (10 g) and polyethylene glycol (PEG) (Sigma-Aldrich, Darmstadt, Germany) (5 g) were stirred for 1 h until complete dissolution. Hexamethylene diisocyanate (HDI) (Sigma-Aldrich, Darmstadt, Germany) was subsequently added in a PEG: HDI molar ratio of 1:4.5. The beginning of polyurethane formation is detected with an increase in viscosity (prepolymer), ending the PU production stage (Figure 1).

**Figure 1:**
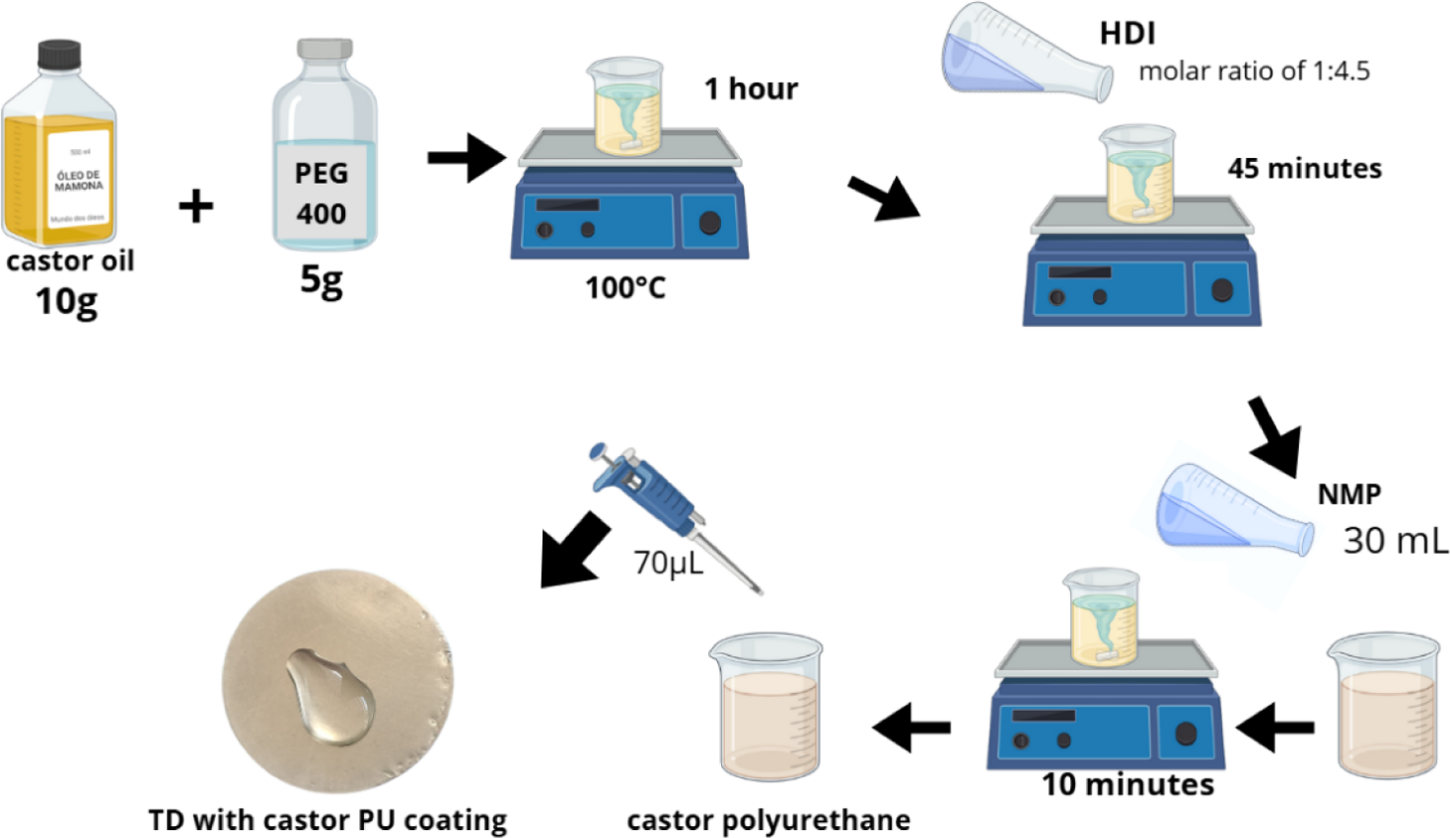
Production of castor oil PU and coating of the titanium disc. All images were created by the authors.

### 2.2 Obtaining and Characterizing Titanium Discs (TDs)

40 samples of grade 4 titanium discs (TDs) were used, with a diameter of 8 mm and 2 mm thickness, produced and donated by the company Titaniumfix (São José dos Campos, SP, Brazil). Ten samples were treated with aluminum oxide blasting and acid etching (SLA).

The samples were divided into three experimental groups, TD with castor PU coating (20), TD with SLA (10), and polished TD (10). The characterization of the polished TD was carried out according to the study by Teixeira et al. (2023),^18^ using water cooling with abrasive paper of 320 to 1200 grains, immersed in acetone, cleaned by ultrasound for 15 min, and after washing with deionized water and 100% ethanol.

### 2.3 Application of the Synthesized Coating

The PU was solubilized in *N*-methyl-2-pyrrolidinone (NMP) (Sigma-Aldrich, Darmstadt, Germany) at 30 mL; the solution was stirred for 10 min to increase the viscosity period. The TDs were coated with 70 μL of the solution, keeping them in a vacuum oven at 65 °C for 2 h (Figure 1).

### 2.4 Characterization of the Titanium Disc with Castor Oil PU

#### 2.4.1 Fourier Transform Infrared Spectroscopy (FTIR)

The FTIR spectrum of the synthesized polyurethane sample was obtained on a Thermo Fisher SCIENTIFIC spectrophotometer, model Nicolet iS5, with a purge pump, wavelength between 400 cm^–1^ and 4000 cm^–1^, in the absorbance module.^12^

Infrared spectroscopy offers a qualitative assessment of the products formed, providing detailed information about the molecular structure through vibrational spectra. These spectra are generated by the absorption of energy during the processes of stretching (axial deformation) and bending (angular deformation) of bonds between atoms of different functional groups. This method was used to verify the completion of the polyurethane curing/synthesis reaction as well as to identify the presence of free isocyanate groups (NCO) after the reaction.

#### 2.4.2 Thermal Analysis

The thermogravimetric analysis (TGA) technique was used to evaluate the stability and thermal decomposition of the polymeric coating obtained as a function of mass loss. Thermal stability was inferred from the TGA curve, as no significant mass loss was observed below 200 °C. The DSC technique was applied to determine the temperature transitions related to the physical state of the polyurethane. The thermal analyzer (TGA-51H, Shimadzu, Japan) was standardized with a heating rate of 10 °C/min; the nitrogen atmosphere was dynamic, with a flow rate of 40 mL/min, up to a temperature of 600 °C and a sample mass of approximately 7 mg.^16^

### 2.5 Characterization of the Experimental Groups (TD with Castor Oil PU, TD with SLA, and Polished TD)

#### 2.5.1 Scanning Electron Microscopy (SEM)

To analyze the surface characteristics of TDs, including their roughness and interconnectivity, three samples (polished TD, TD with SLA, and TD with castor PU) were previously coated with gold, positioned on aluminum stubs, and visualized in SEM (Quanta 250 FEG, FEI, USA). The microscope was operated with an acceleration voltage of 10 kV in the SE mode. The image analysis software ImageJ was used to evaluate the size of the roughness in the images obtained.

#### 2.5.2 Contact Angle

The contact angles (CA) of the TDs were measured by using a charge-coupled device (CCD) camera to capture images and were then calculated with CAM2008 software (KSV Instruments). First, a layer of microparticles was sprayed directly onto the TDs, and then 16 μL drops of ultrapure water (with resistivity greater than 18.2 MΩ × cm) were placed. Each drop was recorded 20 times, and the angle was averaged. CA values were obtained using the mathematical adjustment of the Young/Laplace model.^19^

To evaluate the wettability of the film surface, the sessile drop method was used. This involved carefully depositing a 16 μL droplet of ultrapure water onto the surfaces of the TDs, followed by analyzing the resulting image using CAM 2008 software from KSV Instruments. Each sample had 20 measurements, and the average contact angle was reported.

#### 2.5.3 Atomic Force Microscopy (AFM)

The morphological analysis of the discs was performed by Atomic Force Microscopy, in TT-AFM equipment (AFM Workshop, USA), in intermittent contact mode (vibrating), with a resolution of 512 × 512 pixels, using TAP300-G silicon probes (Ted Pella, USA), with a resonance frequency of approximately 239 kHz. The images were examined using the Gwyddion 2.60 program, from which roughness values (mean roughness, mean quadratic roughness, skewness, kurtosis, and maximum height) were extracted. Representative amplitude, 3D topography, and phase images (5.0 × 5.0 μm) were presented in the results. Statistical analyses were carried out using the GraphPad Prism 8.0.1 program, using two-way ANOVA with Dunnett’s post-test. A *p* < 0.05 was established for statistically significant differences. The results were expressed as mean ± standard error of the mean (SEM).

#### 2.5.4 X-ray Photoelectron Spectroscopic (XPS) Analysis

The chemical analysis of the surface of the TD with castor PU, polished TD, and TD with SLA samples was carried out by X-ray photoelectron spectroscopy (XPS) using a conventional XPS spectrometer (ScientaOmicron ESCA+) with a high-performance hemispherical analyzer (EAC2000) with 128 channels and monochromatic Al Kα radiation (*h* ν = 1486.6 eV) as an excitation source. The operating pressure in the ultrahigh vacuum (UHV) chamber during the analysis was 10–9 Pa. High-resolution XPS spectra were recorded at a constant pass energy of 30 eV with 0.05 eV per step. An electron gun (CN10) was used as a charge neutralizer.

### 2.6 In Vitro Cell Culture Tests

#### 2.6.1 Cell Culture

Cells of the L-929 fibroblast lineage were obtained from the cell bank of the Integrated Center for Morphology and Stem Cell Research (NUPCelt/UFPI). The cells were isolated from clinically healthy mice, characterized by Capella et al. (2019),^20^ cultured in 500 μL of D-MEM F-12 supplemented with 15% fetal bovine serum (FBS) for 24 h.

#### 2.6.2 Cytotoxicity Analysis

L-929 cells were seeded at 2 × 10^5^ cells per well in a 24-well plate and used in this experiment in the 3-(4,5-dimethylthiazol-2-yl)-2,5-diphenyltetrazolium (MTT) assay that evaluates cell viability based on mitochondrial function by reducing MTT bromide to a colored insoluble formazan salt. The TD with castor PU were submerged in the wells of the culture plates and incubated for 24, 48, and 72 h, in triplicate. After that, the samples and culture medium were removed, and the MTT assay was performed. A positive control (L-929 cells in usual culture medium) and a blank were included to validate the viability protocol. Next, an MTT solution (5 mg/mL) was added to the cells to reach a final concentration of 0.5 mg/mL in DMEM and then incubated for 4 h. The medium was removed and replaced with 100 μL of dimethyl sulfoxide (DMSO) to dissolve the formazan crystals. The absorbance of the dyes was recorded at 570 nm on a microplate reader (BioTek Elx 800, Winooski, VT, USA). The number of viable cells after 24, 48, and 72 h was expressed as OD (optical density) of formazan or neutral red dye obtained from cell growth in contact with the control group.

### 2.7 Statistical Analysis

Statistical analyses were performed by two-way ANOVA with GraphPadPrism 8 software for statistical computing. Posthoc comparisons were performed using idák’s posthoc test. Values are expressed as mean ± standard error (S.E.) and were considered significantly different when *p* ≤ 0.05. All data were analyzed and presented using the Statistical Package for the Social Sciences (SPSS), version 20.

## 3 Results

### 3.1 Characterization of Castor Oil PU

Figure 2 shows the infrared spectra of the synthesized PU, in which the infrared energy absorption patterns associated with the chemical groups present in the sample are observed.

**Figure 2:**
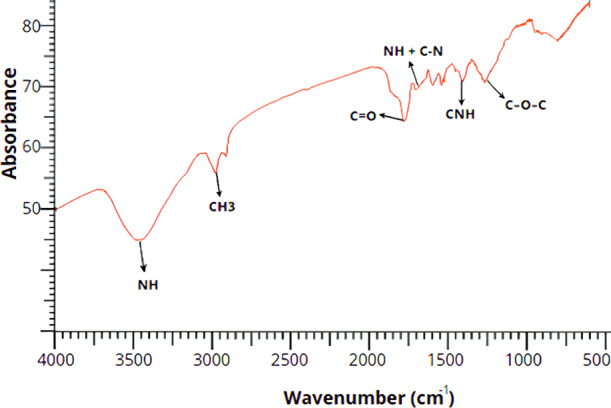
FTIR spectrum of the synthesized castor oil PU. 1- NH stretching; 2- methyl groups (CH_3_); 3- carbonyls (C=O); 4- NH bonds + C–N stretching; 5- presence of driving license; 6- C–O–C bond.

Based on the FTIR test results (Figure 2), the polymer presents an absorption band at 3315 cm-1, which is associated with the NH stretching vibration of urethane (CH_3_OC­(O)­NH_2_.), corroborating the presence of urethane groups. The peaks at 2941 and 2864 cm^–1^ were identified as asymmetric and symmetric vibrations of the methyl groups (CH_3_), respectively. The stretching of the carbonyls (C=O) in the urethane bonds is evidenced at 1681 cm^–1^. The absorption peak at 1541 cm^–1^ corresponds to amide II (NH bonds + C–N stretching), while at 1261 cm^–1^, the presence of CNH is observed, and at 1128 cm^–1^, it refers to the C–O–C bond.

### 3.2 Thermal Analyzes

Figure 3 (Thermogravimetry—TG and Derivative Thermogravimetry—DTG) and Figure 4 (Differential Scanning Calorimetry—DSC), respectively, exemplify the variation in the mass of polyurethane under the same experimental conditions and the flow of thermal energy in relation to temperature. The DSC curve represents the heat flow difference between the polyurethane-coated titanium sample and an empty reference pan as a function of the temperature, allowing the identification of thermal transitions.

**Figure 3:**
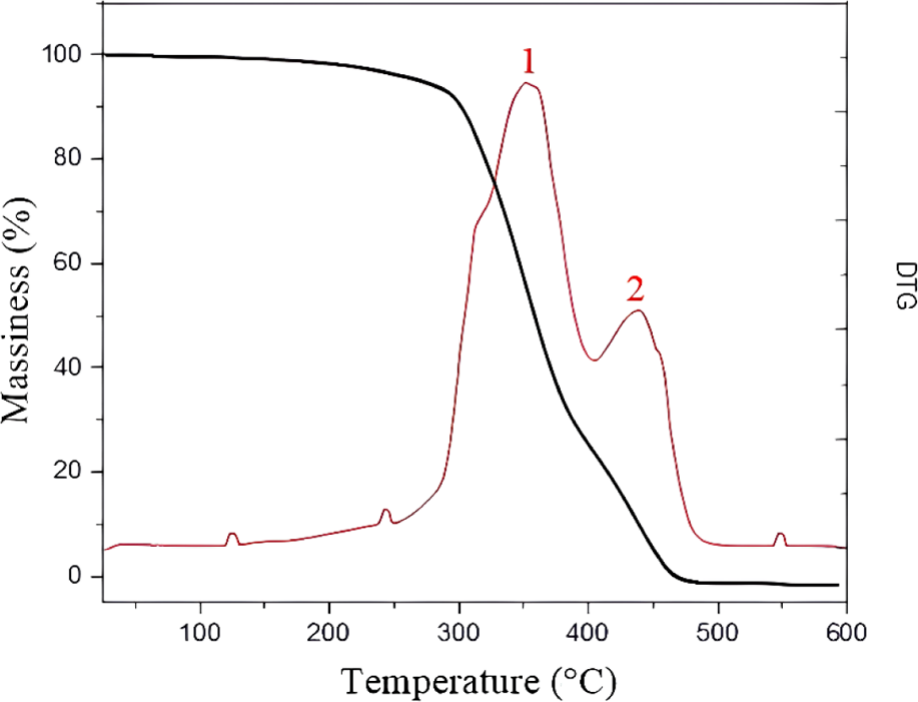
Analysis of TG and DTG of castor oil PU–Decomposition stages.

**Figure 4:**
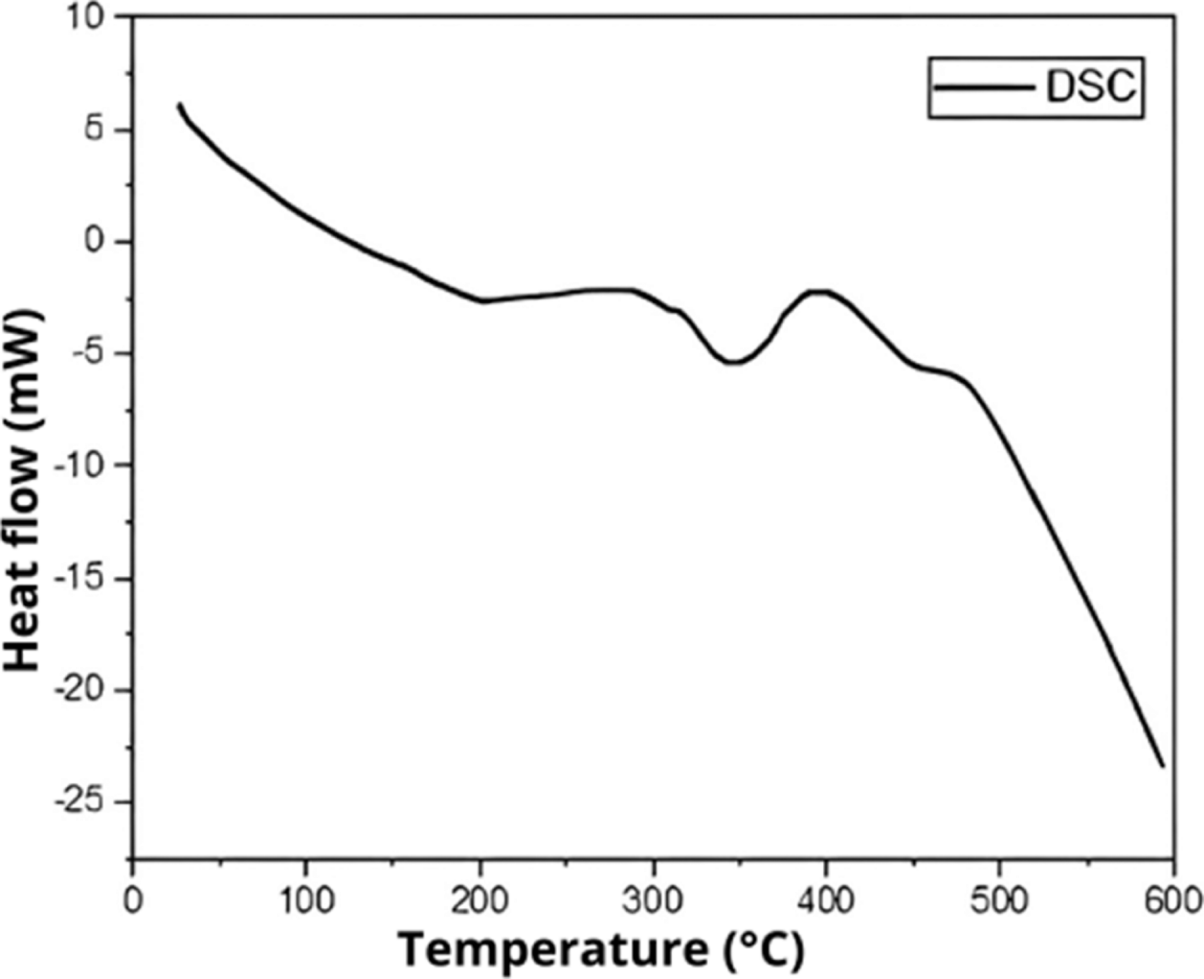
DSC of synthesized castor PU.

The differential thermogravimetry (DTG)—the TGA and DTG curves suggest three main decomposition events: a minor mass loss below 100 °C, attributed to moisture or solvent evaporation, followed by two major events between 300–350 °C and 400–450 °C, corresponding to polyurethane degradation and polymer backbone breakdown, respectively. Around 200 °C, we observed the glass transition of the polymer, noted by the baseline shift in DSC, without significant mass loss in TG. The predominance of the amorphous phase in the biomaterial is indicated by this glass transition without typical melting or recrystallization peaks. Other energy peaks in DSC coincide with mass loss events in TG, such as endothermic peaks around 240 and 250 °C, and exothermic peaks in different temperature ranges, all followed by mass loss. The subsequent loss of 65% of mass between 280 and 400 °C involved the decomposition of fatty acids and the dissociation of polyurethane, followed by the formation of transition components. The remaining mass loss occurred in subsequent steps, characterized by polyol degradation, decomposition of polyurethane functional groups, and thermolysis of organic residues, among other reactions.

#### 3.2.1 Scanning Electron Microscopy (SEM)

Based on the high magnification image, the surface topography of the control sample indicated a smooth structure with grooves demonstrated in the polished TD (Figure 5C). The TD sample with SLA showed nanoscale roughness and granules, confirming its sandblasting on the surface (Figure 5B). The micrograph of the TD with castor oil PU shows defined porosities and sparse grooves, corroborating the characteristics found in other studies, Figure 5A.

**Figure 5:**
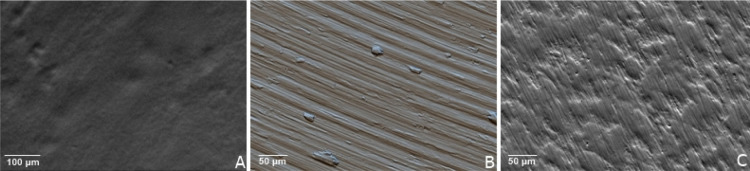
Micrograph: (A) TD with castor oil PU, (B) TD with SLA, and (C) TD polished.

#### 3.2.2 Contact Angle Test

To evaluate the hydrophilic nature of the samples (TD with castor PU, TD with SLA, and polished TD), the water contact angle on their surfaces was determined (Figure 6).

**Figure 6:**
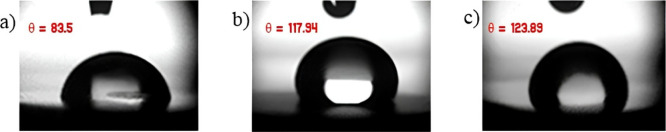
Contact angle: (a) TD with castor oil PU, (b) TD with SLA, and (c) TD polished.

For TDs with castor oil PU, significant differences are observed since the angle values decrease, indicating an increase in the hydrophilic character. The values found for TDs with castor oil PU are close to 90 deg (Table 1).

**Table 1:** Result of the Average Contact Angles

material	degrees (°)
TD with castor oil PU	83.5°
TD with SLA	117.94°
TD polished	123.89°

#### 3.2.3 Atomic Force Microscopy (AFM)

The representative topographies of 5.0 × 5.0 μm demonstrate that the TD with castor oil PU has more uniform and symmetrical roughness of 0.5 μm in thickness when compared to the polished TD and also to the TD with SLA with 0.69 and 0.28 μm, respectively (Figure 7).

**Figure 7:**
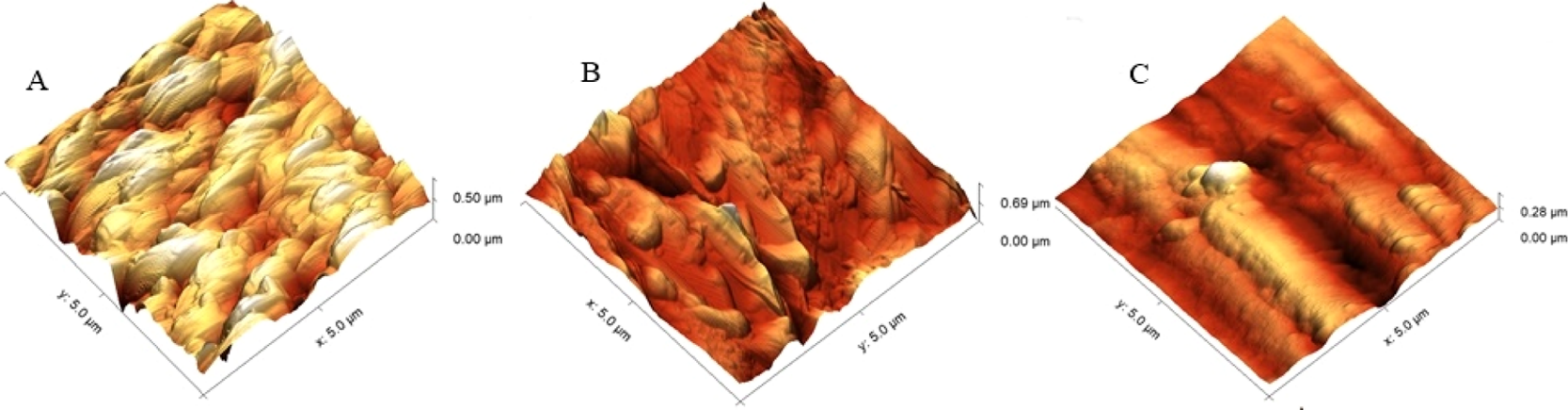
Topographies analyzed by AFM. (A) TD with castor oils, (B) TD with SLA, and (C) TD polished.

#### 3.2.4 X-ray Photoelectron Spectroscopy

The survey spectra for the castor oil, SLA, and polished titanium PU surfaces are observed in Figure 8. C 1s and O 1s signals appear for all of the analyzed TDs. With a similar composition of the TD with SLA and polished TD. Three main constituent elements are present on the polyurethane surface: carbon C 1s at 285 eV, nitrogen N 1s at 399 eV, and oxygen O 1s at 533 eV.

**Figure 8:**
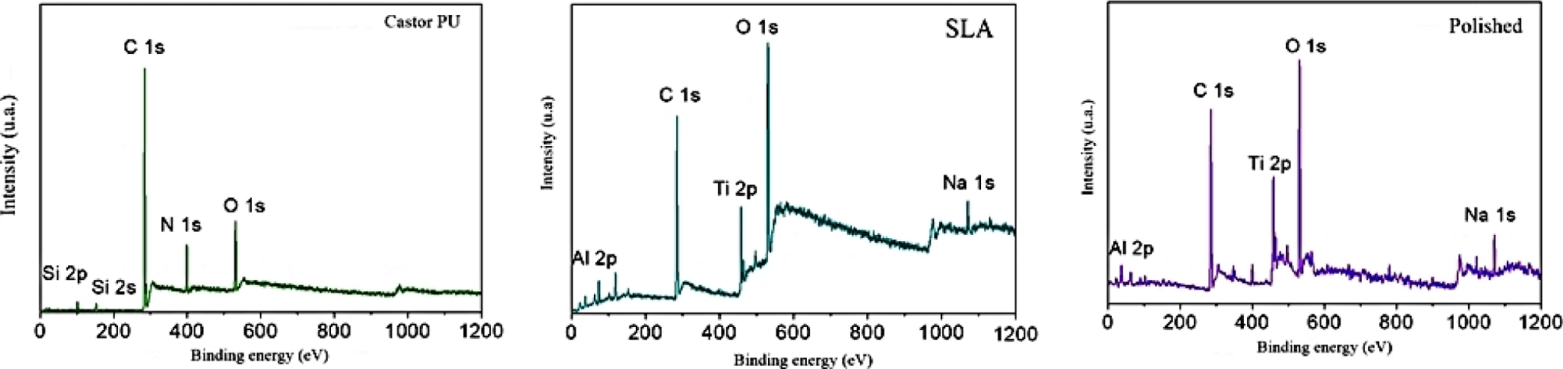
Total spectra of the analyzed discs.

### 3.3 In Vitro Cell Culture Tests

#### 3.3.1 Cytotoxicity Analysis

The analysis was performed in triplicate, with a confidence interval (95% CI = 0.02671 to 0.1086). According to the two-way ANOVA, the mean absorbance shown in Figure 9 (pure titanium = 0.2380; titanium with PU = 0.1703) was close to values related to low cytotoxicity (*p* = 0.0036). L-929 fibroblasts were exposed to extracts of pure titanium (control) and titanium coated with castor oil-based polyurethane (COP) at different times (24 h, 48 h, 72 h). Data represent mean ± standard deviation (*n* = 3). Statistical analysis was performed by two-way ANOVA followed by the Bonferroni posthoc test. ** indicates *p* < 0.01; ns indicates a nonsignificant difference (*p* ≥ 0.05). The horizontal line at 70% viability represents the cytotoxicity threshold according to ISO 10993-5.

**Figure 9:**
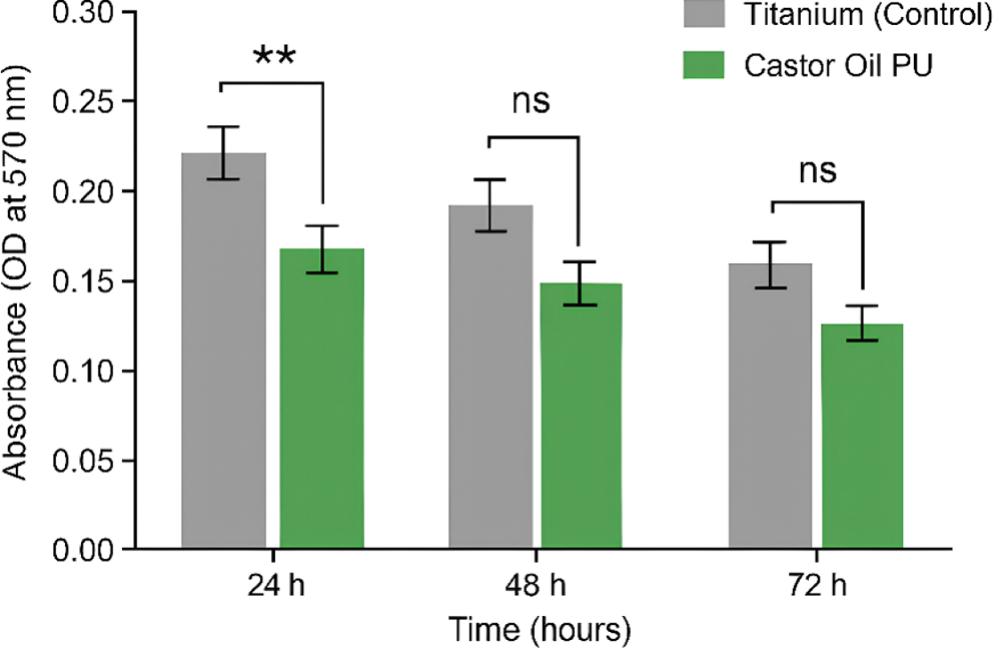
Cell viability of L-929 fibroblasts cultured on titanium (control) and titanium coated with castor oil-based polyurethane (COP) after 24, 48, and 72 h of incubation. Data are expressed as mean ± standard error of the mean (SEM) for triplicate samples. “ns” indicates no significant difference between incubation times (two-way ANOVA, *p* > 0.05). “**” indicates a significant difference between groups (*p* = 0.0036).

## 4 Discussion

Polyurethanes (PU) have been widely explored as biomaterials for the treatment of bone defects due to their low cytotoxicity.^11,14,21^ Titanium, in turn, is widely used in the biomedical and dental fields, especially in oral rehabilitation with dental implants, due to its biocompatibility and nonbiodegradability.^14,21^ However, the high cost of producing titanium dental implants with surface treatments has encouraged the search for more affordable alternatives. In this context, we synthesized polyurethane from a simple reaction using polyol derived from castor oil (*R. communis*), exploiting its synthetic versatility attributed to the high reactivity of the isocyanate group with hydroxyls of compounds such as castor oil.^12,14–16^ This approach not only conserves natural resources but also reduces the environmental impact, offering a more sustainable alternative for the production of polyurethanes.^17^

### 4.1 Physical–Chemical Analysis

FTIR analysis confirmed the efficiency of the synthesis process by identifying functional groups characteristic of castor oil PU and the absence of peaks characteristic of the monomers used. In Figure 2, the infrared spectra of the synthesized PU demonstrate absorption patterns associated with the expected chemical structures. The absence of absorption at 2274 cm^–1^, attributed to isocyanates, indicates that HDI was completely consumed during polymerization, validating its use as a noncytotoxic structure.^14^

Thermal analyses (DSC, TG, and DTG) complemented the characterization, providing data on the thermal stability and degradation behavior of the material. The biomaterial remained stable up to 200 °C, with two thermal decomposition peaks between 300 and 350 and approximately 450 °C. An initial loss of 6% of mass was observed up to 280 °C, related to the evaporation of volatile molecules, as confirmed by the DTG curve (Figure 3). The decomposition steps observed in the TGA/DTG analysis correspond to processes previously reported for castor oil-based polyurethanes, including initial volatilization of low molecular weight compounds and subsequent breakdown of urethane and ester groups.^12–16,21^

The scanning electron microscopy analysis qualitatively revealed a porous surface morphology in the castor oil-based PU coating, characterized by scattered pores and surface irregularities. While no quantitative porosity index was calculated in this study, the morphology suggests a structure that may support cellular migration and osteoconduction. Future studies will aim to quantify porosity and compare it to clinically acceptable ranges.^13,16,18^

### 4.2 Wettability and Topography Analysis

The contact angle values presented in Table 1 highlighted the differences in wettability between the samples. The DT with castor oil PU presented an average of 83.5°, suggesting a hydrophilic behavior (angles <90°), while the TDs with SLA and polished exhibited a hydrophobic character. Studies such as that of Ma et al. (2019)^8^ show that roughness and the degree of hydrophilicity are crucial factors for cell adhesion and proliferation.^9^

The topography was evaluated by AFM, which identified anisotropy on the surface of the TD with castor oil PU. Micropatterns created on the surface simulate the surrounding biological environment, enhancing osseointegration and reducing the risk of inflammation or infection.^22–25^ The Figure 7 revealed directional surface textures on the PU-coated sample, suggesting a potential anisotropic topography. However, the confirmation of anisotropy and presence of a single layer would require more advanced AFM phase imaging or cross-sectional analysis, which were not part of this initial study.^24^

### 4.3 Surface Composition Assessment

XPS analysis revealed significant changes on the surface of the TD with castor oil PU. The relative intensity of the C 1s/N 1s/O 1s elements increased, indicating surface functionalization with groups characteristic of PU. The coating did not alter the internal structure of titanium, preserving its mechanical properties, but it did improve its biological interaction by modifying wettability.^28–31^

### 4.4 Biological Tests

The cytotoxicity assessment by MTT demonstrated that castor oil PU did not trigger major cellular apoptotic behavior with cell viability greater than 70%.^26,27,32^ This suggests the adequate interaction of the material with human tissues without inducing undesirable reactions.^12,13,33–35^ Similar results were reported by Uscátegui et al. (2019),^13^ who highlighted the low toxicity and good mechanical performance of castor oil-based PU.

Complementary studies, such as that of Pacheco et al. (2023),^12^ produced castor oil-based scaffolds with favorable results for cellular activity and bone tissue formation. These findings highlight the potential of castor oil PU both as a surface treatment for cell growth and as a material in tissue engineering.^34^

## 5 Conclusion

Infrared spectroscopy analysis confirmed the efficiency of the synthesis process, while thermal analysis revealed the stability of the polymer over different temperature ranges. SEM and AFM showed roughness and porosity on the polyurethane-coated discs, supporting cell adhesion and osseointegration. Increased surface hydrophilicity, confirmed by contact angle tests, and successful adhesion of polyurethane functional groups identified by XPS highlight the low cytotoxicity of the material. In vitro tests demonstrated low cytotoxicity, with cell viability exceeding 70%.

In conclusion, castor oil-derived polyurethane coatings on titanium exhibited favorable physicochemical and biological properties, including thermal stability, hydrophilicity, cytocompatibility, and surface morphology conducive to cell adhesion. These features make PU a promising candidate for the surface modification of dentistry implants. Further in vivo studies and mechanical testing are warranted to validate the long-term performance and clinical applicability.

## Data Availability

The data supporting the findings of this study are available in the article. Additional information and Supporting Information are available upon request to the corresponding author. Jonathan Ferreira Costa—jonathancosta@ufpi.edu.br.
